# Natural and Synthetic Polymers Modified with Acid Blue 113 for Removal of Cr^3+^, Zn^2+^ and Mn^2+^

**DOI:** 10.3390/polym14112139

**Published:** 2022-05-24

**Authors:** Nicoleta Mirela Marin

**Affiliations:** 1 National Research and Development Institute for Industrial Ecology ECOIND, Street Podu Dambovitei no. 57-73, District 6, 060652 Bucharest, Romania; nicoleta.marin@incdecoind.ro; 2 Science and Engineering of Oxide Materials and Nanomaterials, Faculty of Chemical Engineering and Biotechnologies, University Politehnica of Bucharest, Gh. Polizu 1-7, 011061 Bucharest, Romania

**Keywords:** metal ions, maize stalk, Amberlite XAD7HP, adsorption studies, physical interactions, recovery, chelate reagent, wastewater treatment

## Abstract

This research had two stages of development: during the first stage, the purpose of the research was to evaluate the adsorption properties of the natural polymer represented by shredded maize stalk (MS) and by Amberlite XAD7HP (XAD7HP) acrylic resin for removal of toxic diazo Acid Blue 113 (AB 113) dye from aqueous solutions. The AB 113 concentration was evaluated spectrometrically at 565 nm. In the second stage, the stability of MS loaded with AB 113 (MS-AB 113) and of XAD7HP loaded with AB 113 (XAD7HP-AB 113) in acidic medium suggests that impregnated materials can be used for selective removal of metal ions (Cr^3+^, Zn^2+^ and Mn^2+^). The metal ions using atomic absorption spectroscopy method (AAS) were determined. The use of MS-AB 113 ensures a high selectivity of divalent ions while the XAD7HP-AB 113 had excellent affinity for Cr^3+^ in the presence of Zn^2+^ and Mn^2+^. As a consequence, two advanced polymers, i.e., MS-AB 113 and XAD7HP-AB 113 that provide huge capacity for removal of Zn^2+^, Mn^2+^ and Cr^3+^ from acid polluted wastewater were obtained.

## 1. Introduction

Nowadays, the lack of selectivity of polymeric adsorbents has led to the development of new chelate polymers. In order to obtain a huge selectivity for metal ions (M^X+^) removal new chelate materials have been developed [1,2]. In this, natural and synthetic polymers have proven to be able in preconcentrating and separating M^X+^ [3,4]. Therefore, they contained functional groups that strongly interacted with M^X+^ establishing complex combinations. Many studies have been reported for separation of metal ions from aqueous medium using chelate polymers [5,6]. In this, chelate resins applied for metal ions removal present a multitude of advantages: (i) the selectivity of chelate resin for one and more metal ions (given by the affinity of the ligand immobilized in the resin for M^X+^), (ii) the organic reagent can be removed from the resin and the resin can be recovered and reused and the economical aspect can be taken into consideration; (iii) the impregnation of the resin can be performed in accessible working conditions; (iv) chelate resin has a good stability in acidic medium; and (v) offers a good capacity for M^X+^ removal [7,8,9,10,11,12,13,14,15,16].

The analytical applications for M^X+^ removal from aqueous medium as well as the type of resin used (nature, chemical form of the resin and particle size when is used), organic ligands impregnated in the resin substrate, the M^X+^ retained in the resin as well as the applications of the modified resins are presented in Table 1. As can be observe (Table 1), a wide range of chelate resins with selectivity to metal ions removal from aqueous medium have been used.

The main goal of this research is based on obtaining impregnated natural and acrylic polymers with chelating reagent (AB 113) in order to selectively remove metal ions. Thus, the adsorption mechanism regarding AB 113 binding by MS and XAD7HP, respectively, can be attributed to physical adsorption (π-π interactions) between the aromatic structure of AB 113 and the main components of MS (cellulose, hemicellulose and lignin) and also the alkyl chains of the XAD7HP acrylic resin. In addition, the removal of M^X+^ onto impregnated materials via binding though N donor atoms from the diazo groups of AB 113 structure and Me^x+^ can be established.

The experimental study of this paper consists of: (i) development ecofriendly technologies for AB 113 removal on the natural MS and acrylic polymer XAD7HP; (ii) establishing the optimal conditions for removal of the AB 113 dye from aqueous solutions on MS and XAD7HP; (iii) obtaining new impregnated materials with chelating azo groups (-N=N-) for removal of Cr^3+^, Zn^2+^ and Mn^2+^ from synthetics solutions; and (iv) influence of regeneration studies for selective Cr^3+^, Zn^2+^ and Mn^2+^ recovery from chelate polymers.

## 2. Materials and Methods

### 2.1. Chemicals

The following substances and reagents from Sigma Aldrich and Merck were purchased: XAD7HP resin produced in France, AB 113 (disodium; 8-anilino-5-[[4-[(3-sulfonatophenyl) diazenyl] naphthalen-1-yl] diazenyl] naphthalene-1-sulfonate) and acetone (Ac) from Sigma Aldrich were purchased. The XAD7HP is acrylic ester resin being proper for high molecular organic compounds and metals is only moderately polar. Short information about the characteristics of XAD7HP in the data sheet [17] and in previous research [18] are presented. The ethanol (EtOH) and 37% HCl density 1.16 g/mL (analytical purity without traces of metals) were obtained from Merck. Certified reference materials (CRM) of 1000 mg/L Cr(NO_3_)_3_, Zn(NO_3_)_2_ and Mn(NO_3_)_2_ in 0.5 mol/L HNO_3_ (Merck) were used for the necessary metal ions solutions testing of XAD7HP-AB 113 and MS-AB 113 materials obtained. A 100 mg/L CRM solution containing 21 metallic elements in 3% HNO_3_ of As, Be, Cd, Co, Cr, Co, Cu, Fe, Pb, Li, Mg, Mn, Mo, Ni, Sb, Se, Sr, Tl, Ti, V and Zn (Merck) was used to plot the calibration curves.

### 2.2. Equipment

The UV-Vis spectra of the AB 113 were recorded using the DR/5000 TM spectrometer (Hach Lange, Düsseldorf, Germany) in quartz cuvettes (1 cm) in the 200–800 nm range, with 100 nm/min scanning speed, using ultrapure water as reference.

The PinAAcle 900T atomic absorption spectrometer (Perkin Elmer, Norwalk, CT, USA) was used to determine the concentration of metal ions in the supernatant solution before and after contact with the MS-AB 113 and XAD7HP studied.

The masses of materials (MS, XAD7HP, MS-AB 113, XAD7HP-AB 113) and AB 113 were weighed using the XT220A Precise Gravimetrics scale, Dietikon, Switzerland.

The pH of the supernatant solutions was also measured with the HI 255 pH meter, Hanna Instruments, Nijverheidslaan, Belgium.

To obtained ultrapure water (18 MΩ/cm), an Ultra-Clear system was used (Bremen, Germany).

### 2.3. Preparing the MS for AB 113 Adsorption Experiments

The MS (1-mm particle) swollen in ultrapure water was placed in 2.5 × 25 cm glass column. MS was purified with 4 M HCl solution at 0.6 mL/min flow rate [19]. After acid treatment the MS was washed with ultrapure water until excess acid was removed (pH ≈ 6.5). The MS was then removed from the column, dried at ambient temperature (25 ± 2 °C) in the laboratory for 48 h and subsequently used in adsorption studies for AB 113 removal. The MS with 1-mm particle was obtained as is presented in a previous article [19].

### 2.4. Preparing the XAD7HP Resin for AB 113 Adsorption Experiments

Amberlite XAD7HP resin was added in a Berzelius glass. The resin was washed several times with ultrapure water, decanting the solution until the water became clear. The resin was then allowed to swell in water for 24 h. The swollen resin was transferred to a 2.5 × 25 cm glass column over which a 4 M HCl solution was passed at 0.6 mL/min flow rate [18]. Subsequently, the ultrapure water was passed until pH ≈ 6.5 was obtained. The XAD7HP resin obtained was dried on filter paper in the laboratory for 48 h.

The dried MS and XAD7HP resin were kept in the desiccator throughout the studies.

### 2.5. Procedure for Determining the Dose of Adsorbent Material

To study the influence of MS and XAD7HP dose, 0.25, 0.5 and 1 g were chosen for each material studied. Furthermore, 0.04 L of 100 mg/L AB 113 was added over each sample. The obtained mixtures were stirred for 3 h in the mechanical stirrer horizontally at 175 rpm at 25 ± 2 °C). At the end of the experiment the mixtures were filtered and spectrometrically analyzed.

The quantity of AB 113 adsorption per unit of XAD7HP and MS mass were determined using Equation (1).

Furthermore, efficient removal (R (%)) of AB 113 and M^X+^ on the polymers materials and materials impregnated with AB 113 were evaluated using Equation (2) [20].

All experimental studies were carried out in duplicate and only average values were used for Q_e_ and R (%) determination.

(1) $$Q_{e\;}=\frac{\left(C_{i}-{\;C}_{e}\right)V}{m}\;$$

(2) $$R\;\left(\%\right)=\frac{C_{i}-{\;C}_{e}}{C_{i}}\times 100\;\;$$

where Q_e_ is adsorption capacity, C_i_ and C_e_ (mg/L) are the concentrations of the AB 113 before and after adsorption process, V (L) is the volume of AB 113 added and m (g) are the masses of MS and XAD7HP, respectively.

### 2.6. Batch Adsorption Experiments for AB 113 Removal

Equilibrium adsorption experiments were carried out by introducing 0.25 g of MS and XAD7HP into a 250 mL Erlenmeyer flask. Thus, in each Erlenmeyer flask containing the above quantity mentioned, 0.04 L of AB 113 dye solution was added, having the following concentrations: 100, 113, 125, 138, 150, 163, 176 and 189 mg/L, respectively. The mixtures thus obtained were stirred for 3 h at 175 rpm using the mechanical stirrer in the horizontal plane at 25 ± 2 °C. After stirring, the dye-loaded materials were separated on filter paper and the filtrates were collected in volumetric flasks. The spectrum of each filtered solution was recorded and the amount of AB 113 in the filtrate was determined spectrometrically. All experimental studies were carried out in duplicate and for Q_e_ determination the average values obtained were taken into consideration.

### 2.7. Procedure for Recovery AB 113 from MS and XAD7HP

To study the influence of organic agents for AB 113 recovery from impregnated materials—MS-AB 113 and XAD7HP-AB 113—obtained when equilibrium was reached (20.9 mg AB 113/g of MS and 25 mg AB 113/g of XAD7HP resin), samples of 0.25 g of each material loaded were weighed. Then, 0.04 L of EtOH and Ac were added. The obtained mixtures were stirred for 60 min in the mechanical stirrer horizontally at 175 rpm (25 ± 2 °C). At the end of the experiment each mixture was filtered and spectrometrically analyzed. All experimental studies were carried out in duplicate and the average value obtained for AB 113 recovery from MS and XAD7HP of each experiment was used.

### 2.8. Characterization of Materials Loaded in the Presence of Acidic Solutions

The MS-AB 113 and XAD7HP-AB 113 obtained were characterized in terms of stability in the acid environment by proceeding as follows: 0.25 g MS-AB 113 and XAD7HP-AB 113 were stirred for 60 min with 0.04 L acidic solutions of 1–2 M HCl. Each sample of material at the end of the stirring time was filtered on quantitative filter paper. All filtration solutions were collected in volumetric flasks and the concentration of AB 113 was spectrometrically determined. All experimental studies were carried out in duplicate and the average value obtained for AB 113 recovery from MS and XAD7HP in each experiment was used.

### 2.9. Metal Ions Removal Procedures

To obtained impregnated materials, four samples of 0.25 g XAD7HP and MS were impregnated with 40 mL of 163 mg/L AB 113 at 175 rpm (25 ± 2 °C). At the end of stirring time the solid phases of MS-AB 113 and XAD7HP-AB 113 materials were obtained. Furthermore, 0.25 g of individually impregnated materials were stirred with 40 mL of M^X+^ solution with 0.15 mg/L (S1), 0.25 mg/L (S2), 0.35 mg/L (S3) and 0.45 mg/L (S4), respectively, for 60 min at 175 rpm (25 ± 2 °C). At the end of the experiment each solution was analyzed by AAS method for M^X+^ determination. All experimental studies were carried out in duplicate and for M^X+^ (%) on MS-AB 113 and XAD7HP-AB 113 the average value obtained was taken into consideration.

### 2.10. Procedure for Recovery of M^X+^ from the MS-AB 113 and XAD7HP-AB 113

The MS-AB 113 and XAD7HP-AB 113 obtained after removal solid metal phases—S1-M^X+^, S2-M^X+^, S3-M^X+^ and S4-M^X+^—were regenerated as follows: 0.25 g of solid phase corresponding to each material was stirred for 60 min with 0.04 L acidic solutions (2 M HCl) at 175 rpm at 25 ± 2 °C. At the end of the stirring time, each mixture was filtered on quantitative filter paper. All filtration solutions were collected in volumetric flasks and the concentration of M^X+^ released via AAS method were determined. All experimental studies were carried out in duplicate and for M^X+^ (%) released from (MS-AB113)-M^X+^ and (XAD7HP-AB 113)-M^X+^ into liquid phases the average value obtained was taken into consideration.

### 2.11. Preparation of Metal Ions Solutions to Establish the Linearity of the Atomic Absorption Spectrometric Method (AAS)

To determine the linearity of the AAS method, 5 standard solutions from 21 metallic elements CRM (stock solution) of 100 mg/L were prepared. The linearity of the method was checked in the 0.1–0.5 mg/L range for each metal ion. For this purpose, a volume of 5 mL MRC solution was passed into a 50 mL volumetric flask and a working solution of 10 mg/L was obtained. Subsequently the solutions for checking the linearity were obtained by normality law: 0.1, 0.2, 0.3, 0.4 and 0.5 mg/L. Each sample was read at the following wavelengths (λ): 279.48 nm Mn^2+^, 357.87 nm Cr^3+^ and 213.86 nm Zn^2+^, respectively. The values used to draw the calibration curves represent the average of three determinations. The calibration curves were automatically drawn for each metal ion (representing the A = f(C)) by the equipment software. The equations of the calibration curves and correlation coefficient (R^2^) were: y = 0.2996x + 0.0042 with R^2^ = 0.999 for Zn^2+^; y = 0.0839x − 0.0035, R^2^ = 0.9995 for Mn^2+^; and y = 0.0098x − 0.0003, R^2^ = 0.9992 for Cr^3+^.

### 2.12. Spectrometric Determination of AB 113

**Figure 1:**
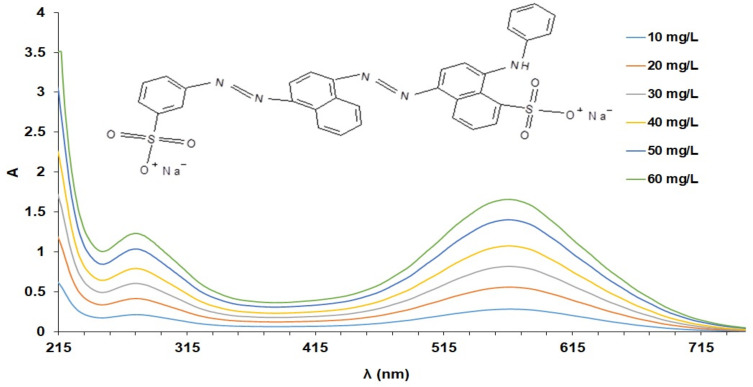
Representation of A = f (λ) for 10, 20, 30, 40, 50 and 60 mg/L solutions of AB 113.

This study presents the results of spectrometric experiments in order to establish the optimal working conditions for AB 113 determination. To record the spectra A = f (λ), AB 113 solutions obtained from 0.1 g/L AB 113 stock solution by dilution in 10 mL volumetric flasks were used. As can be seen in Figure 1, the AB 113 spectrum has two maxima at 275 and 565 nm at which the absorbance can be read. For AB 113 determination the calibration curve was obtained by graphical representation A = f(C), where A is the absorbance read at λ = 565 nm. Concentration (C) of AB 113 solutions for linearity of method were: 10, 20, 30, 40, 50 and 60 mg/L. The linearity concentrations in the wavelength range of 200–800 nm were recorded. The obtained spectra are presented in Figure 1. After fitting the experimental data, Beer’s law was checked for all concentration ranges. The equation of the calibration curves A = 0.0274 C + 0.0064 with R^2^ = 0.9990 was also obtained.

### 2.13. Determination of LD and LQ for UV-VIS Spectrometric Method

The limit of detection (LD) is defined as the lowest concentration of analyte in the sample studied, which can be identified, but cannot be quantified [21,22]. The equation for LD is:

LD = 3s(3)

LD for AB 113 determining in aqueous solution was calculated by applying Equation (3), for ten blank samples (ultrapure water of 18 MΩ/cm) recorded at λ = 565 nm.

Where s is the standard deviation of the blank samples (µg/L). The s for blank samples was calculated as being 0.5 µg/L. The LD was 1.5 µg/L AB 113.

The limit of quantification (LQ) is defined as the lowest analyte concentration in the sample, which can be determined quantitatively, at an acceptable level of uncertainty [21,22]. The equation used was:

LQ = 10s(4)

The LQ for AB 113 applying Equation (4) was determined as being LQ = 5 µg/L AB 113.

### 2.14. Determination of LD and LQ for AAS Method

To determine LDs and LQs for Mn^2+^, Zn^2+^ and Cr^3+^, signal intensity for 10 blank samples (ultrapure water of 18 MΩ/cm) at 279.48 nm for Mn^2+^, 357.87 nm for Cr^3+^ and at 213.86 nm for Zn^2+^ were recorded. The s (µg/L) for blank samples were calculated as being 0.13 µg/L Mn^2+^, 0.2 µg/L Zn^2+^ and 0.23 µg/L Cr^3+^, respectively, and by applying Equations (3) and (4) the LDs and LQs for Cr^3+^, Zn^2+^ and Mn^2+^ were obtained [22]. The results obtained for LDs and LQs of Mn^2+^, Zn^2+^ and Cr^3+^ are shown in Table 2.

## 3. Results and Discussion

### 3.1. Influence of MS and XAD7HP Dosage

**Figure 2:**
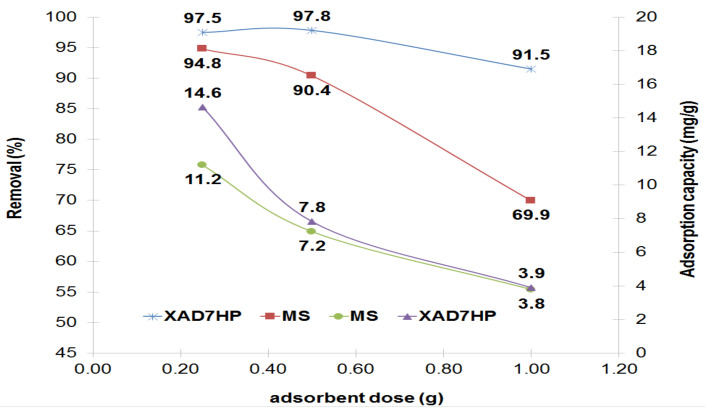
Effect of MS and XAD7HP dosage on AB 113 removal, Ci = 100 mg/L AB 113, pH = 8.04 for MS supernatant and pH = 7.83 for XAD7HP.

Literature data suggest that the dose of material significantly influences the adsorption process [23,24]. The rapid adsorption process is economically improved if the dose of material is correctly chosen. The influence of MS and XAD7HP dose on the AB 113 removal is illustrated in Figure 2. It was observed, that with increasing the dosage of MS and XAD7HP from 0.25 to 0.5 and up to 1 g, the highest value of Q_e_ (mg/g) at 0.25 g for both materials tested was obtained. Then, the value of Q_e_ (mg/g) decreases to 7.2 mg/g for MS and 7.8 mg/g for XAD7HP. Furthermore, when 1 g of MS and XAD7HP was used for AB 113 adsorption, the Q_e_ up to 3.8 mg/g for MS and 3.9 mg/g of XAD7HP decreased. Taking into account this observation, a dosage of 0.25 g of MS and XAD7HP can be used for efficient AB 113 removal. Thus, 0.25 g was used in all experimental studies to optimize the adsorption conditions for AB 113 removal. Similar results on dosage effect of dye removal by natural (NWS) and modified wheat straw (MWS) and also by activated carbon (AC) were obtained by Mousa et al. The capacity (mg/g) of NWS, MWS and AC decreased by increasing dosage amount by applying the same conditions regarding concentration, pH, volume of dye and temperature [25]. Another study regarding the influence of dosage for Cr^6+^ removal from wastewater by magnetite nanoparticles was evaluated. To establish optimum adsorbent dosage, an experimental study was done. The magnetite nanoparticles were increased from 5 to 10 g/L. The obtained results suggested that with the increasing dose of material, agglomeration of the particles takes place. Maximum Cr^6+^ removal at 6 g/L was obtained for the 5–10 g/L doses studied [26].

### 3.2. Influence of AB 113 Concentrations on the MS and XAD7HP Resin

**Figure 3:**
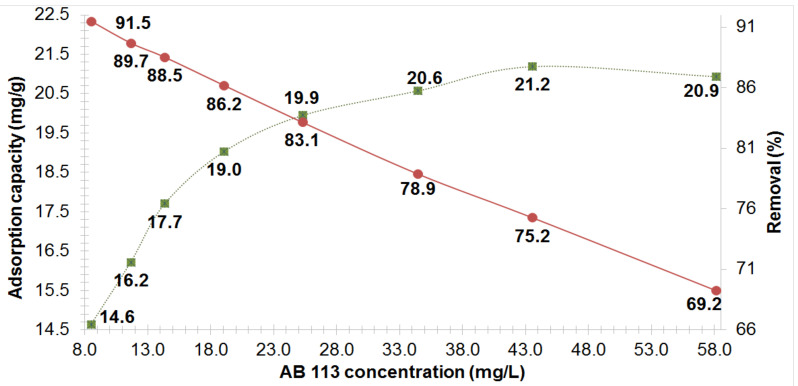
Effect of AB 113 initial concentration on the removal (%) and the adsorption capacity of MS adsorbent.

**Figure 4:**
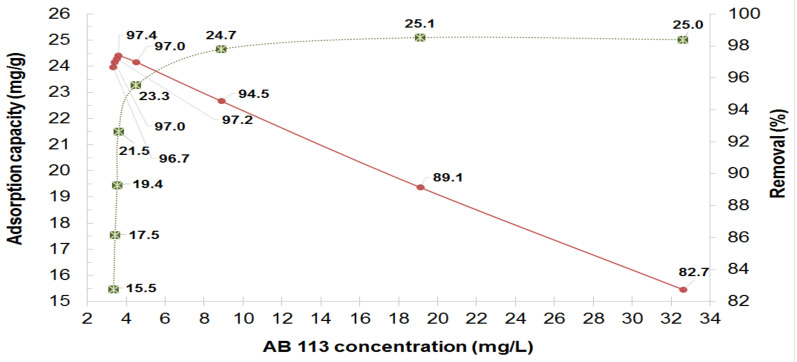
Effect of AB 113 initial concentration on the removal (%) and the adsorption capacity of XAD7HP adsorbent.

The adsorption proprieties of MS and XAD7HP resin with AB 113 was studied according to the initial dye concentration. In order to obtain a complete image of the adsorption process when initial concentrations (C_i_) varied from: 100, 135, 125, 138, 150, 163 and 176 up to 189 mg/L, Equation (1) was applied. The results obtained for adsorption capacity of MS and XAD7HP are shown in Figure 3 and Figure 4.

The efficient removal R (%) of adsorption process by Equation (2) was done. The R (%) results determined for MS and XAD7HP for AB 113 adsorption are shown in Figure 3 and Figure 4.

It was observed that with the increase of AB 113 initial concentrations tested, an increase of AB 113 dye amount retained on the MS and XAD7HP mass is obtained. The Q_e_ of AB 113 adsorption by MS mass at equilibrium were: 14.6, 16.2, 17.7, 19, 19.9, 20.6, 21.2 and 20.9 mg/g, respectively. It was found that at higher concentrations than 150 mg/L of AB 113 in the initial solution, the quantity of AB 113 removed onto 0.25 g MS varies insignificantly. Taking into account these observations, Q_e_ was obtained at 20.9 mg/g for MS. For the XAD7HP acrylic resin applying the same procedures, the Q_e_ (mg/g) values increased from 15.5 17.5, 19.4, 21.5, 23.3, 24.7, 25.1 up to 25 mg/g for AB 113 concentrations of 100, 113, 125, 138, 150, 163, 176 and 189 mg/L. It can be concluded that the adsorption capacity of XAD7HP resin for AB 113 adsorption (25 mg/g) was higher than for MS (20.9 mg/g) using the same experimental conditions.

The following studies have been performed for dye removal using XAD7HP resin. According to the results obtained by Kerkez et al., XAD7HP resin was tested for methylene blue (MB) adsorption. The Q_m_ (maximum adsorption capacity) was 2.72 mg/g determined by Langmuir isotherm. Furthermore, 21.55 mg/g was obtained using the same conditions for XAD-16 [27]. Marin et al. removed AB 113 from aqueous solutions by batch technique onto Amberlite IRA 402 (Cl^−^). For this, 0.5 g of dried IRA 402 (Cl^−^) was stirred for 90 min with 50 mL of 2.07 g/L AB 113, where the Qe was 117 mg/g [28]. Moreover, several publications reported AB 113 adsorption using low-cost adsorbents. Peyman et al. investigated AB 113 adsorption onto a composite obtained from activated carbon (AC)-ZnO nanoparticles (ZnO). For adsorption experiments, 0.5 g of AC-ZnO was stirred together with 100 mg/L AB 113, with 15 min reaction time. By previous conditions presented, 98.2% AB 113 was removed. [29]. By using activated red mud (ARM) the AB 113 and Reactive Black 5 (RB5) removal was investigated. The Q_m_ was 83.33 mg AB 113/g ARM and 35.58 mg RB5/g ARM [30]. Thus, 128 mg AB 113/g of C-Fe_2_O_3_ was obtained (where C-Fe_2_O_3_ is chitosan magnetized by Fe_2_O_3_ nanoparticles) [31].

### 3.3. Influence of Organic Agent on AB 113 Desorption

**Figure 5:**
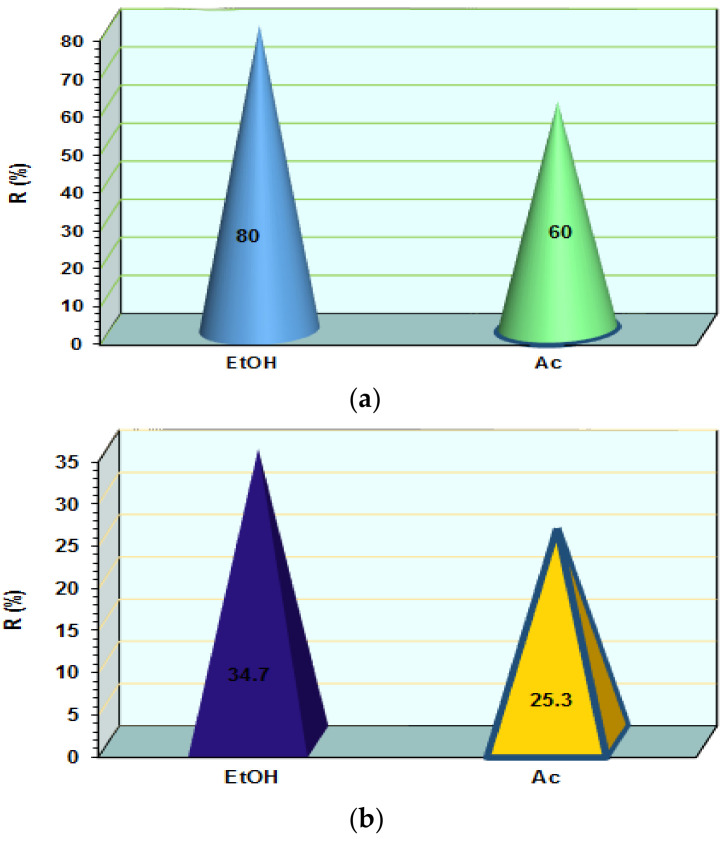
Effect of EtOH and Ac on the AB 113 recovery from XAD7HP (**a**) and MS (**b**).

The EtOH and Ac on the regenerate MS-AB 113 and XAD7HP-AB 113 materials were used. The regeneration experiment was performed by the batch method taking into account recommendations from the technical data sheet of XAD7HP. The XAD7HP-AB 113, after regeneration with EtOH, released 80% AB 113 in supernatant from its mass (Figure 5a). Furthermore, 60% AB 113 was detected in the supernatant when Ac was used for XAD7HP-AB113 regeneration (Figure 5a). In order to regenerate the MS-AB 113, the same organic agents were used. When EtOH was used for AB 113 desorption, MS-AB 113 in supernatant released only 34.7% AB 113 and 65.3% AB 113 was still loaded in the MS mass (Figure 5b). Furthermore, when Ac was used for MS-AB 113 regenerations, 74.7% AB 113 remained loaded in the MS mass and only 25.3% AB 113 was detected in the supernatant (Figure 5b). Thus, for the regeneration of polymers loaded with AB 113, EtOH can be a better choice than Ac.

Soto et al., proposed the efficient desorption study of phenolic compounds from polymeric resin, i.e., XAD2, XAD4, XAD7HP, XAD16HP, XAD761 and XAD1180 with ethanol [32]. Furthermore, in a previously study, XAD7HP loaded with PHA and LID was efficiently regenerated with Ac where 82% PHA and 75% LID is detected in liquid phases [18]. Furthermore, good yield of IMPXD7 (XAD7-HP impregnated with Aliquot 336) loaded with Reactive Blue 13 regenerations were obtained when EtOH was used [24].

### 3.4. Influence of Acid Medium on AB 113 Desorption

The stability of XAD7HP–AB 113 and MS-AB 113 resins using 1–2 M HCl were assessed. In this, the mechanism of stability was analyzed by procedure proposed for determination of AB 113 in acidic solutions. By recording the molecular absorption spectra of the filtrate solutions, it was found that the supernatant acid solutions contained dye in very low percentages. Only 2.6% AB 113 was found in 1 M HCl solutions and 3.1% in 2 M solutions for XAD7HP-AB 113, while for MS-AB 113, 3.5 and 5% AB 113 were found. To conclude, the stability of loaded materials is slightly influenced by acidic environment solutions. According to the results obtained by Marin et al., the AB 113 recovery from IRA 402 anionic resin using 1–7 M HCl solutions was evaluated. The percentage of AB 113 in liquid phases increased in acidic solutions with increase of HCl concentration. The AB 113 remained in IRA 402 mass up to 2 M HCl solutions [28]. Furthermore, in another study, Marin et al. reported acidic influence by Amberlite IRA 400 (IRA 400) resin loaded with Acid Orange 10 (AOG 10). The results obtained showed that up to 1 M HCl, resin loaded with azo dye is stable [33]. Moreover, the IRA 400 stability loaded with Acid Blue 193 (AB 193) was studied in basic (0.1–0.01 M NaOH) and acidic mediums (0.1–0.01 M HCl). The AB 193 dye detected in liquid phases was below LQ of the analytical method [34].

### 3.5. Applications of Loaded Polymers for Aqueous Samples Depollution with Cr^3+^, Zn^2+^ and Mn^2+^

**Figure 6:**
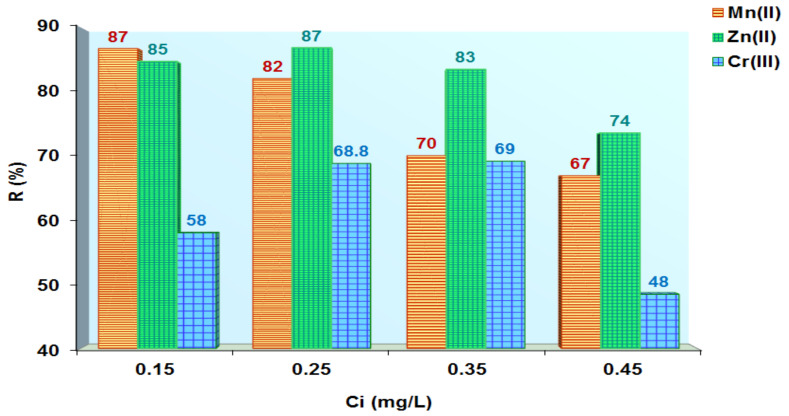
Metal ions removed from aqueous solutions using MS-AB 113.

The new materials (MS-AB 113 and XAD7HP-AB 113) obtained were applied as an alternative for heavy metal removal from aqueous solutions. Therefore, MS-AB 113 and XAD7HP-AB 113 were used by four different aqueous solutions having 0.15 mg/L (S1), 0.25 mg/L (S2), 0.35 mg/L (S3) and 0.45 mg/L (S4) of Cr^3+^, Zn^2+^ and Mn^2+^. For S1 concentration, the metal ion removal onto MS-AB 113 was observed as being 87% Mn^2+^ followed by 85% Zn^2+^ and by 58% Cr^3+^. Thus, it is observed that MS-AB 113 removed over 50% of the studied metal ions for the S2 concentration, S3 concentration and S4 concentration. The highest percent removal was observed for divalent metals ions for all S1–S4 concentrations studied, while Cr^3+^ was slightly removed (Figure 6).

**Figure 7:**
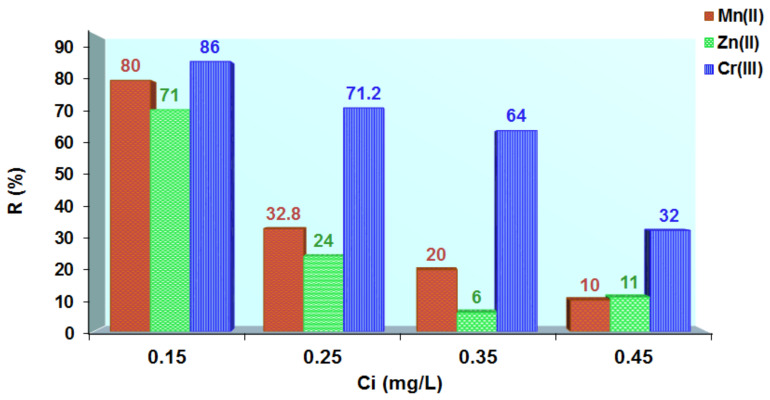
Metal ions removed from aqueous solutions using XAD7HP-AB 113.

The XAD7HP-AB 113 removed the highest percent all metal ions studied (86% Cr^3+^, 80% Mn^2+^ and 71% Zn^2+)^ for S1 concentration. Then, at S2 concentration, Cr^3+^ removal (%) is double that of Mn^2+^ but triple that of Zn^2+^. At S3 concentration, the Cr^3+^ removal (%) is ten times that of Zn^2+^ and three times that of Mn^2+^. Lastly, at S4 concentration, the Cr^3+^ removal (%) is almost threefold that of both Mn^2+^ and Zn^2+^ ions (Figure 7).

In a previous study, Marin et al. reported that the PHA (4-amino-N-[2-(diethylamino) ethyl] benzamide) and LID (2-Diethylamino-N-(2,6-dimethylphenyl) acetamide), were used for XAD7HP impregnations. The new materials obtained, i.e., XAD7HP-PHA and XAD7HP-LID, were tested for As^5+^, Cd^2+^ and Pb^2+^ removal. From the synthetic mixture investigated, XAD7HP-PHA has a good affinity for As^5+^ while XAD7HP-LID efficiently removed Pb^2+^. The excellent metal removal properties of this impregnated polymer provide a secondary alternative to improve water depollution with toxic metals represented by As^5+^ and Pb^2+^ [18]. Furthermore, literature review studies emphasized that the XAD7HP impregnated with organic reagents has high affinity for Cr^3+^ removal [35,36]. Cipec et al. studied Cr^3+^ removal from aqueous solutions by the impregnated Amberlite XAD7 resin with di-(2-ethylhexyl)-phosphoric acid (DEHPA). For Cr^3+^ removal, the Q_m_ was 3 mg/g for C_i_ of 10 mg Cr^3+^/L at pH 3 and contact time of 90 min [35]. Davidescu et al. used impregnated XAD7 resin with DEHPA as extractant and EtOH as organic solvent to obtained the support of material. To establish the optimal condition of adsorption the influence of experimental parameters was investigated. The Q_m_ was 5 mg Cr^3+^/g of impregnated material when C_i_ varied from 5 to 40 mg Cr^3+^/L [36]. Thus, Zheng et al. reported that the cellulose extracted from corn stalk was modified with -CN and -OH groups in order to improve capacity of the raw material. The adsorption capacity of cellulose modified for Cd^2+^ removal was 21.37 mg/g compared to 3.81 mg/g unmodified raw corn stalk [37]. The chelating resin obtained by cellulose when glycine is immobilized on it (CELL-GLY) has been used for pre-concentrations Cu^2+^ and Ni^2+^. The method can be applied for metals determination in trace amounts [38].

### 3.6. Desorption of Metal Ions from the Loaded Polymers

**Figure 8:**
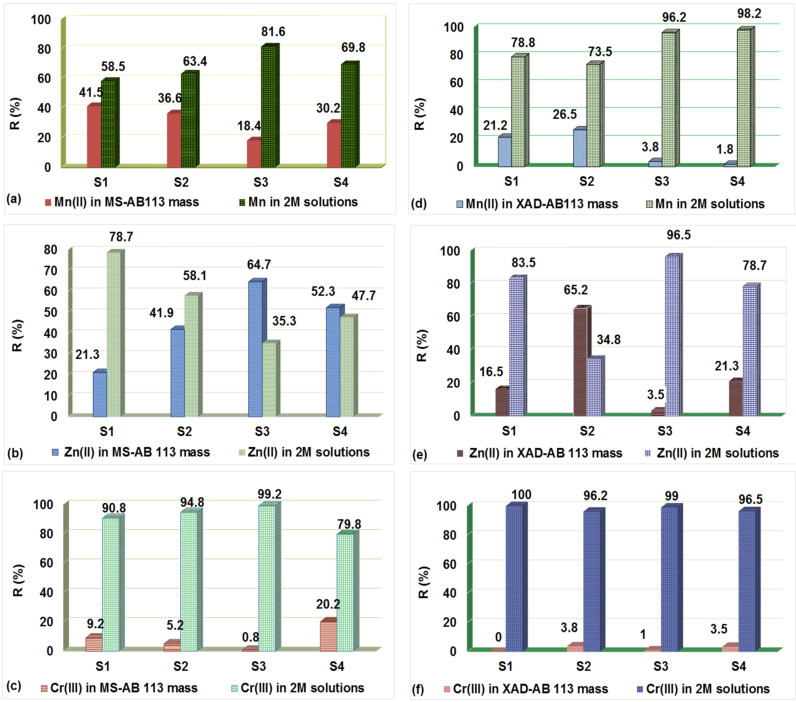
Effect of HCl solutions on the metal ions desorbed by: MS-AB 113 (**a**–**c**) and XAD7HP-AB 113 (**d**–**f**).

The following hypothesis for the metal ions recovery from MS-AB and 113 XAD7HP–AB 113 can be taken into consideration: (i) by the influence of the regeneration agent the metallic ion (M^X+^) is released into solution in the form of AB 113-M^X+^ if the AB 113 with M^X+^ forms a stabile complex; or (ii) under the action of the regeneration agent are released in solution only M^X+^ and AB 113 still remaining loaded in the mass of material [28]. In this, the aim was to recover only metal ions. As can be seen in Figure 8a–c the 2 M HCl solution released most of the metal ions retained on MS-AB 113.

For XAD7HP-AB 113 resin loaded with M^X+^, the behavior of acid action is in accordance with the affinity of the XAD7HP-AB 113 for retaining the metal ions. Thus, the best metal ions retained (Cr^3+^) released the most in 2 M HCL solution. Moreover, the Mn^2+^ and Zn^2+^ existing in solid samples were also desorbed by the 2 M HCl—Figure 8d–f.

Exhausted maize stalk with Cu^2+^ and Pb^2+^ was regenerated with acidic solutions (3 M HNO_3_). Furthermore, 93% Cu^2+^ and 89% Pb^2+^ was recovered from maize stalk mass in liquid phases [19]. Furthermore, it was detected that 63% Cu^2+^ and 89% Fe^3+^ are released from maize stalk mass with 4 M HCl [39]. The chemical stability of metal ions, i.e., As^5+^, Cd^2+^, Co^2+^, Cr^3+^, Fe^3+^, Mn^2+^, Mo^6+^, Ni^2+^, Pb^2+^, Sb^3+^, V^2+^, Zn^2+^, Tl^3+^ and Sr^2+^ retained onto maize stalk with 1 and 4 M HCl was investigated. The influence of acidic solutions obeyed the theoretical rules when 4 M HCl was used; the majority of metal ions were released in liquid phases [40]. As presented by Mittal et al. elution of Cu^2+^ and Ni^2+^ from CELL-GLY was evaluated with 0.1 M HCl and more than 95% of each metal was recovered [38].

## 4. Conclusions

In this research, eco-friendly materials were employed for removal of the first pollutant (AB 113), and subsequently to remove secondary pollutants (metal ions). The optimum dose for efficient AB 113 removal was chosen to be 0.25 g for MS and XAD7HP by proposed procedure. It was also observed that the MS and XAD7HP have good adsorption capacity for AB 113. EtOH was used as optimal solvent for AB 113 desorption from both polymeric materials studied. In addition, only 5% AB 113 was found in 2 M HCl liquid phases of MS-AB 113. The XAD7HP-AB 113 under 2 M HCl acidic action released 3.1% AB 113. All these results suggested that MS-AB 113 and XAD7HP-AB 113 have chemical stability in acidic medium up to 2 M HCl. The MS-AB 113 and XAD7HP-AB 113 materials having chelating properties were obtained by physical π-π interactions. It was proved that the MS-AB 113 and XAD7HP-AB 113 are relatively easy to obtain. Based on those results, MS-AB 113 and XAD7HP-AB 113 can be recommended for removal of Cr^3+^, Zn^2+^ and Mn^2+^ from acidic polluted wastewaters. Furthermore, following the desorption studies, the removed metals can be recovered by applying the ecological processes developed in this study. Taking into account capacity and selectivity, this article proposed polymeric materials, i.e., XAD7HP resin and MS for azo dye and polymers loaded with AB 113 to metal ions removal. Furthermore, the XAD7HP polymer resin can be a good choice if the acquisition cost is comparatively higher with MS. On the other hand, MS is easy to obtain (found in abundance at the global level) and represents a sustainable material for ecofriendly technologies.

## Figures and Tables

**Table 1:** Applications of chelating resin for metal ions removal.

Resin Type/Chemical Form/Particle Size	Chelating Agents	Metal Ion	Applications	References
Amberlite IRA 400, in Cl^−^ form, 20–25 mesh (600–750 μm)	Cresol Red	Hg^2+^, Fe^3+^, Cu^2+^, Al^3+^ and Ni^2+^	Hg^2+^ was separated by the Fe^3+^, Cu^2+^, Al^3+^ and Ni^2^	Khan et al. [7]
Amberlite IRC-50, in COO^−^ form, 16–50 mesh size	Thiosemicarbazone	Au^3+^ and Ag^+^ separation from binary mixtures	Au^3+^ and Ag^+^ were separated by the Cu^2+^ and Pb^2+^	Roy et al. [8]
D001-strongly acidic cation exchange resin, in SO_3_^−^ form 0.40 to 0.85 mm,	Polyethylenimine (PEI) and glutaraldehyde	Cu^2+^, Mg^2+^, Ca^2+^, Sr^2+^,	Cu^2+^ was separated in the presence of Mg^2+^, Ca^2+^, Sr^2+^	Chen et al. [9]
Amberlite XAD-4, polystyrene divinyl benzene, 20–50 mesh	8-hydroxy quinoline	U^4+^	Synthetic solutions	Singh et al. [10]
Amberlite IRA 402, in Cl^−^ form, 20–60 mesh	2-(*p*-Sulfophenylazo)-1,8-dihydroxy-3,6-naphthalene disulfonate (SPADNS)	Cu^2+^, Co^2+^, Cd^2+^, Ni^2+^, Mn^2+^ and Fe^3+^	Synthetic solutions	Wawrzkiewicz et al. [11]
Amberlite XAD-16, styrene divinylbenzene, 20–60 mesh	1-(2-pyridylazo) 2-naphtol	Ni^2+^, Cd^2+^, Co^2+^, Cu^2+^, Pb^2+^ and Cr^3+^	Natural water	Narin et al. [12]
Amberlite XAD-4, styrene divinylbenzene, 20–60 mesh	Tri Octyl Phosphine Oxide (Cyanex 921)	La^3+^	Wastewater	Fatah et al. [13]
Sulfonated polystyrene (PSN)	Polyethyleneimine (PEI) and dopamine (PDA)	Pb^2+^, Fe^3+^, Cu^2+^ and Ni^2+^	Wastewater	Gao et al. [14]
Cellulose acetate (CA)	Polyethyleneimine grafting (CAP) and then by ethylenediamine (CAPE)	Cu^2+^ and Pb^2+^	Synthetic solutions	Huang et al. [15]
Biochar	Chitosan and pyromellitic dianhydride (PMDA)	Cd^2+^, Cu^2+^ and Pb^2+^	Synthetic solutions	Deng et al. [16]

**Table 2:** Validation parameters of the AAS method.

Parameter	Mn(II)	Zn(II)	Cr(III)
LD (µg/L)	0.4	0.6	0.7
LQ (µg/L)	1.3	2.0	2.3

## Data Availability

The data presented in this study are available on request from the corresponding author.
